# Ischemic Placental Disease and Severe Morbidity in Pregnant Patients With Sleep Disorders

**DOI:** 10.1001/jamanetworkopen.2025.32189

**Published:** 2025-09-16

**Authors:** Naima Ross, Rebecca J. Baer, Scott P. Oltman, Dana R. Gossett, R. Nisha Aurora, Laura Jelliffe-Pawlowski, Justin S. Brandt

**Affiliations:** 1 Division of Maternal-Fetal Medicine, Department of Obstetrics and Gynecology, NYU Grossman School of Medicine, New York, New York; 2 NYU Langone Health, New York; 3 Department of Pediatrics, University of California San Diego, La Jolla; 4 The California Preterm Birth Initiative, University of California San Francisco; 5 Department of Obstetrics, Gynecology and Reproductive Sciences, University of California San Francisco; 6 Department of Biostatistics and Epidemiology, University of California San Francisco School of Medicine; 7 Institute of Global Health Sciences, University of California San Francisco School of Medicine; 8 Department of Obstetrics and Gynecology, NYU Grossman School of Medicine, New York, New York; 9 Department of Medicine, NYU Langone Health, New York; 10 Rory Meyers College of Nursing, NYU, New York

## Abstract

**Importance:**

Insomnia and obstructive sleep apnea (OSA) are associated with pregnancy complications.

**Objective:**

To evaluate the association of insomnia and OSA during pregnancy with the risk of ischemic placental disease (IPD) and severe morbidity (SM) and to compare these risks between the 2 sleep disorders.

**Design, Setting, and Participants:**

This cross-sectional study included a statewide population-based sample of liveborn singleton births with linked birth certificates for birthing people and their infants in California from January 1, 2011, through December 31, 2020. The analysis was performed on July 22, 2024.

**Exposures:**

Insomnia and OSA.

**Main Outcomes and Measures:**

The main outcomes were IPD, defined as preeclampsia, placental abruption, and birth of a neonate small for gestational age (SGA), and SM, defined according to the Centers for Disease Control and Prevention definition.

**Results:**

During the study period, there were 4 145 096 singleton live births among birthing people aged 13 to 55 years; 4783 (0.1%) had insomnia, 5642 (0.1%) had OSA, and 4 134 671 (99.7%) had neither condition. The prevalence of insomnia and OSA was 116 and 136 cases per 1000 live births, respectively. Compared with patients without insomnia or OSA (738 660 [17.9%]), the adjusted relative risk (ARR) of any IPD was 1.42 (95% CI, 1.35-1.50) for those with insomnia (1406 patients [29.4%]) and 1.57 (95% CI, 1.50-1.64) for those with OSA (1848 [32.8%]). Compared with patients with neither disorder, the ARR of birth of an SGA neonate was higher for those with insomnia (1.23; 95% CI, 1.13-1.35) than for those with OSA. The ARR of preterm birth was 1.81 (95% CI, 1.68-1.95) for insomnia (711 patients [14.9%]) and 1.73 (95% CI, 1.62-1.85) for OSA (870 [15.4%]) vs neither disorder (279 364 [6.8%]). The ARR of SM was 2.26 (95% CI, 2.03-2.50) for insomnia (366 patients [7.7%]) and 2.81 (95% CI, 2.58-3.06) for OSA (545 [9.7%]) vs neither disorder (93 857 [2.3%]).

**Conclusions and Relevance:**

In this cross-sectional study of singleton live births, pregnant individuals with insomnia or OSA were at increased risk for IPD, SM, and preterm birth compared with those without these sleep disorders. Further study is needed to determine the biological mechanisms for these risks and whether early identification and targeted preventive interventions may improve pregnancy outcomes.

## Key Points

**Question:**

What is the risk of ischemic placental disease and severe morbidity among pregnant patients with insomnia or obstructive sleep apnea (OSA)?

**Findings:**

In this cross-sectional study of 4 145 096 pregnant individuals with singleton live births, the adjusted risk of ischemic placental disease and severe morbidity in patients with insomnia or OSA was higher than in those without a sleep disorder. Insomnia and OSA were associated with an increased risk of preterm birth.

**Meaning:**

The findings suggest that insomnia or OSA during pregnancy may be associated with increased risk of ischemic placental disease, severe morbidity, and preterm birth.

## Teaser

This cross-sectional study examines associations of insomnia and obstructive sleep apnea during pregnancy with risk of ischemic placental disease and severe morbidity.

## Introduction

Sleep disorders during pregnancy are common, affecting nearly 70% of pregnant people, and can significantly affect the health of the birthing parent and fetus.^1,2,3,4^ Hormonal fluctuations and physiologic changes, such as weight gain, urinary frequency, and increased respiratory drive, are associated with sleep disturbances.^3,5^ Obstructive sleep apnea (OSA), insomnia, and restless leg syndrome have been associated with adverse pregnancy outcomes, including preeclampsia, gestational diabetes, perinatal depression, preterm birth, and pregnancy-related morbidity and mortality.^6,7,8,9^ OSA has been associated with severe morbidity (SM) and ischemic placental disease (IPD), a syndrome that includes preeclampsia, placental abruption, and fetal growth restriction.^10,11,12,13,14^

While much of the literature on sleep disorders in pregnancy has focused on OSA, insomnia is the most common sleep disorder, and the prevalence increases throughout pregnancy and the postpartum period.^15,16,17^ In the nonpregnant population, insomnia is associated with decreased immune function and cardiovascular disease.^18^ Insomnia is a well-established risk factor for perinatal mood disorders, specifically depression, anxiety, and suicidal ideation, and has also been associated with preterm birth.^19,20,21,22^ Literature suggests that insomnia in pregnancy disproportionally affects racial and ethnic minority individuals and people with low socioeconomic status.^15^ Despite the burden of insomnia on peripartum health, there are limited data available to guide counseling, recognition, and treatment.

We performed a population-based cross-sectional study of singleton live births in California to evaluate the associations between insomnia and risk of IPD and SM and to compare the magnitude of this risk with that associated with OSA. We hypothesized that insomnia, like OSA, would be associated with both IPD and SM and that our results would underscore the need for improved recognition and treatment of insomnia in pregnancy.

## Methods

We performed a population-based cross-sectional study of singleton live births in California from January 1, 2011, through December 31, 2020. Methods and protocols for the study were approved by the Committee for the Protection of Human Subjects within the Health and Human Services Agency of the State of California. Informed consent was waived by the State of California because data were deidentified and there was low risk to participants. Per Department of Health Care Access and Information (HCAI) requirements, no cell sizes with fewer than 11 individuals are presented. Birth certificates, maintained by California Vital Statistics, were linked to maternal and infant hospital discharge, emergency department, and ambulatory surgery records maintained by the HCAI.^23^ Hospital discharge, emergency department, and ambulatory surgery files provided diagnoses and procedure codes based on the *International Classification of Diseases, Ninth Revision (ICD-9)* and *International Statistical Classification of Diseases and Related Health Problems, 10th Revision (ICD-10)*, as reported to the HCAI by health care facilities.^24^ As such, these diagnoses were derived from clinical encounters and based on reporting within the medical records. The study sample was restricted to singleton live births with linked birth records for birthing people and their infants (Figure). This study followed the Strengthening the Reporting of Observational Studies in Epidemiology (STROBE) reporting guideline for cross-sectional studies.

**Figure.**
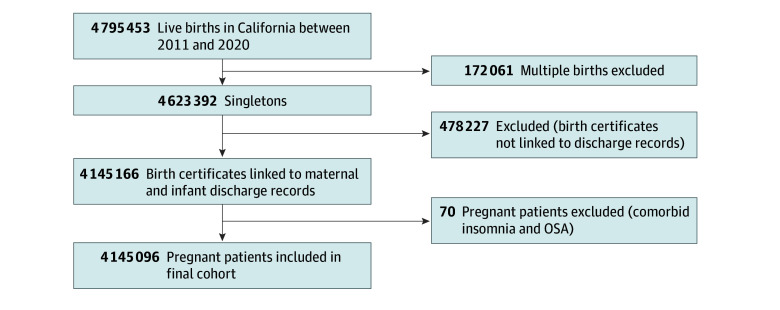
Study Flow Diagram OSA indicates obstructive sleep apnea.

Diagnoses of insomnia and OSA (eTable 1 in Supplement 1) were obtained from HCAI records during pregnancy or at the birth admission. Various subcategories of insomnia were included in the *ICD-9* and *ICD-10* codes, including both acute and chronic insomnia. IPD included hypertensive disorders of pregnancy (including preeclampsia with and without severe features), placental abruption, and birth of a neonate small for gestational age (SGA) (eTable 1 in Supplement 1). SGA was defined as birth weight less than the 10th percentile for sex and gestational age.^25^ Sex and gestational age of neonates were obtained from birth certificate records. Gestational age (by completed week) was reported based on best obstetric estimate. Preterm birth was defined as birth occurring at less than 37 weeks’ gestation and was subgrouped as gestational ages of less than 28 weeks, 28 to less than 32 weeks, 32 to less than 34 weeks, and 34 to less than 37 weeks. The US Centers for Disease Control and Prevention definition of severe maternal morbidity was used, and diagnoses were obtained from birth certificates and HCAI *ICD-9* and *ICD-10* diagnostic and procedure codes.^26^

Maternal factors considered from birth certificate records included age at delivery, race and ethnicity (Asian, Hispanic, non-Hispanic Black, non-Hispanic White, or other [American Indian or Alaska Native, Native Hawaiian or Pacific Islander, multiracial, or unknown or not stated]), expected payer for delivery (private, Medi-Cal, or other), educational level (<12 years, 12 years, or >12 years), prepregnancy body mass index (BMI; calculated as weight in kilograms divided by height in meters squared), country of birth, and parity. Mode of delivery, tobacco smoking during pregnancy, hypertension without preeclampsia, and diabetes were obtained from both birth certificate and HCAI records. Depression during pregnancy was obtained from HCAI records (eTable 1 in Supplement 1). Race and ethnicity, included in the analysis to assess potential disparate and inequitable outcomes, were assumed to be self-reported on the birth certificate.

### Statistical Analysis

The relative risk (RR) of IPD or preterm birth among people with insomnia or OSA was calculated using Poisson log-linear regression, with people without insomnia or OSA and no IPD as the reference populations. The RR was calculated for IPD or preterm birth overall and by contributing diagnoses and gestational age subgroups. RRs were adjusted for maternal age, payer, educational level, prepregnancy BMI, parity, country of birth, diabetes, chronic hypertension, gestational hypertension, perinatal depression, and tobacco smoking during pregnancy.

The RR of SM among people with insomnia or OSA was calculated using Poisson log-linear regression with people without insomnia or OSA and no SM as the reference populations. The RR was calculated for SM overall, SM without blood transfusions, and for each of the 21 diagnoses that define SM.^26^ RRs were adjusted for maternal age, payer, educational level, prepregnancy BMI, parity, country of birth, diabetes, chronic hypertension, gestational hypertension, perinatal depression, and tobacco smoking during pregnancy.

While our primary focus was not on comparing outcomes in those with insomnia vs those with OSA (focusing instead on the comparison of risks associated with these conditions vs no sleep disorders), we included an analysis comparing the risks of IPD and SM in patients with insomnia vs OSA (eTables 2 and 3 in Supplement 1). All analyses were performed using SAS, version 9.4 (SAS Institute Inc). The analysis was performed on July 22, 2024.

## Results

During the study period, there were 4 145 096 singleton live births among birthing people aged 13 to 55 years with linked birth certificate and hospital records, of whom 4783 (0.1%) had insomnia, 5642 (0.1%) had OSA, and 4 134 671 (99.7%) had neither condition (Figure). Of the total population, 14.6% were Asian, 48.7% were Hispanic, 4.8% were non-Hispanic Black, 26.4% were non-Hispanic White, and 5.5% were other race and ethnicity. The prevalence of insomnia and OSA was 116 and 136 cases per 1000 live births, respectively. Demographic characteristics and medical comorbidities are described in Table 1. A total of 77.2% of the population without insomnia or OSA was 18 to 34 years of age, while 3316 (69.3%) of those with insomnia and 3275 (58.1%) of those with OSA were 18 to 34 years of age. Among patients with insomnia, 352 (7.4%) were Asian, 1605 (33.6%) were Hispanic, 383 (8.0%) were non-Hispanic Black, 2018 (42.2%) were non-Hispanic White, and 425 (8.9%) were other race and ethnicity. Overall, 2861 (59.8%) were privately insured, 2929 (61.2%) had vaginal deliveries, 2964 (62.0%) had more than 12 years of education, 2050 (42.9%) had a BMI between 18.5 and less than 25, 650 (13.6%) smoked tobacco during pregnancy, and 1408 (29.4%) had depression. Among those with an OSA diagnosis, 632 (11.2%) were Asian, 2161 (38.3%) were Hispanic, 615 (10.9%) were non-Hispanic Black, 1646 (29.2%) were non-Hispanic White, and 588 (10.4%) were other race and ethnicity. Overall, 4001 (70.9%) had a BMI of 30 or greater, 371 (6.6%) smoked tobacco during pregnancy, and 947 (16.8%) had depression. There was minimal overlap between sleep disorder groups; only 70 of 10 425 patients with sleep disorders (0.7%) had comorbid insomnia and OSA and were excluded from the study.

**Table 1.** Maternal Demographics by Sleep Disorder During Pregnancy in a Cross-Sectional Study of Singleton Live Births in California, 2011-2020

Characteristic	Patients, No. (%)		
	No insomnia or OSA (n = 4 134 671)	Insomnia (n = 4783)	OSA (n = 5642)
Age at delivery, y			
<18	61 545 (1.5)	39 (0.8)	23 (0.4)
18-34	3 193 377 (77.2)	3316 (69.3)	3275 (58.1)
>34	879 634 (21.3)	1428 (29.9)	2344 (41.6)
Race and ethnicity			
Asian	603 771 (14.6)	352 (7.4)	632 (11.2)
Hispanic	2 013 601 (48.7)	1605 (33.6)	2161 (38.3)
Non-Hispanic Black	200 004 (4.8)	383 (8.0)	615 (10.9)
Non-Hispanic White	1 092 356 (26.4)	2018 (42.2)	1646 (29.2)
Other^a^	224 939 (5.4)	425 (8.9)	588 (10.4)
Expected payer for delivery			
Private	2 002 713 (48.4)	2861 (59.8)	3854 (68.3)
Medi-Cal	1 863 731 (45.1)	1729 (36.2)	1628 (28.9)
Other	268 227 (6.5)	193 (4.0)	160 (2.8)
Mode of delivery			
Vaginal	2 828 831 (68.4)	2929 (61.2)	2454 (43.5)
Cesarean	1 236 372 (29.9)	1839 (38.5)	3181 (56.4)
Educational level, y			
<12	655 931 (15.9)	461 (9.6)	398 (7.1)
12	1 007 214 (24.4)	1110 (23.2)	1213 (21.5)
>12	2 279 758 (55.1)	2964 (62.0)	3630 (64.3)
Prepregnancy BMI			
<18.5	150 172 (3.6)	163 (3.4)	36 (0.6)
18.5 to <25	1 838 502 (44.5)	2050 (42.9)	592 (10.5)
25 to <30	1 057 141 (25.6)	1201 (25.1)	840 (14.9)
≥30	940 245 (22.7)	1220 (25.5)	4001 (70.9)
Country of birth			
US	2 454 760 (61.6)	3863 (80.8)	4540 (80.5)
Other	1 588 911 (38.4)	920 (19.2)	1102 (19.5)
Nulliparous	1 602 152 (38.8)	1937 (40.5)	2171 (38.5)
Smoked tobacco during pregnancy	116 513 (2.8)	650 (13.6)	371 (6.6)
Chronic hypertension without preeclampsia	60 508 (1.5)	200 (4.2)	674 (12.0)
Preexisting diabetes	50 457 (1.2)	131 (2.7)	570 (10.1)
Gestational diabetes	411 983 (10.0)	609 (12.7)	1413 (25.0)
Depression	92 621 (2.2)	1408 (29.4)	947 (16.8)

Abbreviations: BMI, body mass index (calculated as weight in kilograms divided by height in meters squared); OSA, obstructive sleep apnea.

^a^ Included American Indian or Alaska Native, Native Hawaiian or Pacific Islander, multiracial, or unknown or not stated.

Compared with people with no sleep disorders (738 660 [17.9%]), the adjusted RR (ARR) of any IPD was 1.42 (95% CI, 1.35-1.50) for those with insomnia (1406 patients [29.4%]) and 1.57 (95% CI, 1.50-1.64) for those with OSA (1848 patients [32.8%]) (Table 2). Compared with people with neither sleep disorder, the risk for placental abruption was elevated for people with insomnia and for those with OSA, whereas the risk for birth of an SGA neonate was elevated only for people with insomnia (ARR, 1.23; 95% CI, 1.13-1.35). The ARR of any preterm birth at less than 37 weeks’ gestation was 1.81 (95% CI, 1.68-1.95) for insomnia (711 patients [14.9%]) and 1.73 (95% CI, 1.62-1.85) for OSA (870 [15.4%]) vs neither disorder (279 364 [6.8%]). The ARR of preterm birth at less than 28 weeks’ gestation was 2.42 (95% CI, 1.81-3.23) for those with insomnia and 2.10 (95% CI, 1.61-2.75) for those with OSA vs those with neither sleep disorder, and the ARR of preterm birth from 28 to less than 32 weeks’ gestation was 3.11 (95% CI, 2.53-3.82) for those with insomnia and 2.47 (95% CI, 2.02-3.03) for those with OSA. The ARR for preeclampsia was 2.26 (95% CI, 2.13-2.40) in those with OSA and 1.84 (95% CI, 1.68-2.01) in those with insomnia compared with those with neither sleep disorder.

**Table 2.** Risk of IPD and PTB by Sleep Disorder in a Cross-Sectional Study of Singleton Live Births in California, 2011-2020

Outcome	Patients, No. (%)	RR (95% CI)	ARR (95% CI)^a^
No IPD or PTB			
No insomnia or OSA	3 396 011 (82.1)	NA	NA
Insomnia	3377 (70.6)	NA	NA
OSA	3794 (67.3)	NA	NA
Any IPD^b^			
No insomnia or OSA	738 660 (17.9)	1 [Reference]	1 [Reference]
Insomnia	1406 (29.4)	1.64 (1.56-1.73)	1.42 (1.35-1.50)
OSA	1848 (32.8)	1.83 (1.75-1.92)	1.57 (1.50-1.64)
Any preeclampsia^b^			
No insomnia or OSA	173 229 (4.2)	1 [Reference]	1 [Reference]
Insomnia	492 (10.3)	2.62 (2.40-2.86)	1.84 (1.68-2.01)
OSA	1126 (20.0)	4.72 (4.45-5.00)	2.26 (2.13-2.40)
Preeclampsia with severe features^b^			
No insomnia or OSA	93 247 (2.3)	1 [Reference]	1 [Reference]
Insomnia	357 (7.5)	3.58 (3.22-3.97)	2.31 (2.08-2.57)
OSA	857 (15.2)	6.90 (6.45-7.37)	2.86 (2.67-3.06)
Preeclampsia without severe features^b^			
No insomnia or OSA	85 651 (2.1)	1 [Reference]	1 [Reference]
Insomnia	168 (3.5)	1.93 (1.66-2.24)	1.46 (1.26-1.70)
OSA	329 (5.8)	3.24 (2.91-3.61)	1.77 (1.59-1.97)
Placental abruption			
No insomnia or OSA	40 194 (1.0)	1 [Reference]	1 [Reference]
Insomnia	97 (2.0)	2.39 (1.96-2.91)	1.83 (1.50-2.23)
OSA	102 (1.8)	2.24 (1.84-2.72)	1.83 (1.50-2.22)
Birth of SGA neonate^c^			
No insomnia or OSA	359 678 (8.7)	1 [Reference]	1 [Reference]
Insomnia	497 (10.4)	1.33 (1.23-1.46)	1.23 (1.13-1.35)
OSA	397 (7.0)	0.99 (0.90-1.09)	1.07 (0.97-1.18)
Preterm birth, by gestational age			
All <37 wk			
No insomnia or OSA	279 364 (6.8)	1 [Reference]	1 [Reference]
Insomnia	711 (14.9)	2.29 (2.13-2.46)	1.81 (1.68-1.95)
OSA	870 (15.4)	2.45 (2.30-2.62)	1.73 (1.62-1.85)
<28 wk			
No insomnia or OSA	14 639 (0.4)	1 [Reference]	1 [Reference]
Insomnia	47 (1.0)	3.20 (2.40-4.26)	2.42 (1.81-3.23)
OSA	55 (1.0)	3.33 (2.55-4.34)	2.10 (1.61-2.75)
28 to <32 wk			
No insomnia or OSA	21 380 (0.5)	1 [Reference]	1 [Reference]
Insomnia	93 (1.9)	4.28 (3.49-5.25)	3.11 (2.53-3.82)
OSA	97 (1.7)	3.98 (3.26-4.86)	2.47 (2.02-3.03)
32 to <34 wk			
No insomnia or OSA	27 063 (0.7)	1 [Reference]	1 [Reference]
Insomnia	96 (2.0)	3.50 (2.86-4.27)	2.55 (2.08-3.12)
OSA	133 (2.4)	4.28 (3.61-5.08)	2.75 (2.32-3.27)
34 to <37 wk			
No insomnia or OSA	216 282 (5.2)	1 [Reference]	1 [Reference]
Insomnia	475 (9.9)	2.06 (1.88-2.25)	1.65 (1.51-1.81)
OSA	585 (10.4)	2.23 (2.06-2.42)	1.61 (1.48-1.75)

Abbreviations: ARR, adjusted relative risk; IPD, ischemic placental disease; NA, not applicable; OSA, obstructive sleep apnea; PTB, preterm birth; RR, relative risk; SGA, small for gestational age.

^a^ Adjusted for maternal age, payer, educational level, prepregnancy body mass index, parity, country of birth, diabetes, chronic hypertension, gestational hypertension, perinatal depression, and tobacco smoking during pregnancy.

^b^ Not adjusted for hypertension disorders.

^c^ Birth weight less than the 10th percentile, per Talge et al.^25^

Compared with those with without a sleep disorder (93 857 patients [2.3%]), the ARR of any SM was 2.26 (95% CI, 2.03-2.50) for insomnia (366 patients [7.7%]) and 2.81 (95% CI, 2.58-3.06) for OSA (545 patients [9.7%]) (Table 3). The comparative risk of SM was even greater for nontransfusion SM (insomnia: ARR, 2.74 [95% CI, 2.40-3.12]; OSA: 3.76 [95% CI, 3.40-4.16]), with 231 patients with insomnia (4.8%) and 394 with OSA (7.0%) having a nontransfusion SM. Compared with patients without either sleep disorder, the ARR was higher for patients with insomnia than it was for those with OSA for the following SMs: disseminated intravascular coagulation (2.38 [95% CI, 1.68-3.37] vs 2.27 [95% CI, 1.62-3.19]), puerperal cerebrovascular disorders (4.23 [95% CI, 2.67-6.70] vs 2.64 [95% CI, 1.55-4.49]), sepsis (2.66 [95% CI, 2.10-3.37] vs 2.65 [95% CI, 2.11-3.34]), shock (3.61 [95% CI, 2.31-5.63] vs 3.36 [95% CI, 2.13-5.31]), air or thrombotic embolism (3.72 [95% CI, 2.36-6.13] vs 3.25 [95% CI, 2.07-5.08]), and hysterectomy (3.02 [95% CI, 1.81-5.04] vs 2.46 [95% CI, 1.52-3.98]). The ARRs for all other components of SM were greater in people with OSA than in those with insomnia compared with people with neither sleep disorder.

**Table 3.** Risk of Severe Maternal Morbidity by Sleep Disorder in a Cross-Sectional Study of Singleton Live Births in California, 2011-2020^a^

Outcome	Patients, No. (%)	RR (95% CI)	ARR (95% CI)^b^
No severe morbidity			
No insomnia or OSA	4 040 814 (97.7)	NA	NA
Insomnia	4417 (92.4)	NA	NA
OSA	5097 (90.3)	NA	NA
Any severe morbidity			
No insomnia or OSA	93 857 (2.3)	1 [Reference]	1 [Reference]
Insomnia	366 (7.7)	3.37 (3.04-3.74)	2.26 (2.03-2.50)
OSA	545 (9.7)	4.26 (3.91-4.63)	2.81 (2.58-3.06)
Severe morbidity without blood transfusions			
No insomnia or OSA	45 419 (1.1)	1 [Reference]	1 [Reference]
Insomnia	231 (4.8)	4.47 (3.93-5.09)	2.74 (2.40-3.12)
OSA	394 (7.0)	6.46 (5.85-7.13)	3.76 (3.40-4.16)
Acute kidney failure			
No insomnia or OSA	4969 (0.1)	1 [Reference]	1 [Reference]
Insomnia	28 (0.6)	5.12 (3.54-7.44)	2.48 (1.70-3.61)
OSA	53 (0.9)	8.38 (6.39-10.98)	3.50 (2.65-4.61)
Acute respiratory distress syndrome			
No insomnia or OSA	4571 (0.1)	1 [Reference]	1 [Reference]
Insomnia	50 (1.1)	9.91 (7.50-13.09)	4.96 (3.73-6.60)
OSA	85 (1.5)	14.52 (11.71-17.99)	7.19 (5.76-8.97)
Disseminated intravascular coagulation			
No insomnia or OSA	10 200 (0.3)	1 [Reference]	1 [Reference]
Insomnia	32 (0.7)	2.86 (2.02-4.04)	2.38 (1.68-3.37)
OSA	34 (0.6)	2.63 (1.88-3.69)	2.27 (1.62-3.19)
Blood transfusion			
No insomnia or OSA	46 876 (1.1)	1 [Reference]	1 [Reference]
Insomnia	142 (3.0)	2.72 (2.30-3.20)	2.10 (1.78-2.48)
OSA	175 (3.1)	2.89 (2.50-3.36)	2.29 (1.97-2.66)
Eclampsia			
No insomnia or OSA	6379 (0.2)	1 [Reference]	1 [Reference]
Insomnia	26 (0.5)	3.71 (2.53-5.46)	2.68 (1.82-3.95)
OSA	35 (0.6)	4.33 (3.10-6.03)	3.09 (2.21-4.33)
Puerperal cerebrovascular disorders			
No insomnia or OSA	2104 (0.1)	1 [Reference]	1 [Reference]
Insomnia	19 (0.4)	8.23 (5.24-12.93)	4.23 (2.67-6.70)
OSA	14 (0.3)	5.26 (3.11-8.90)	2.64 (1.55-4.49)
Pulmonary edema and/or acute heart failure			
No insomnia or OSA	3246 (0.1)	1 [Reference]	1 [Reference]
Insomnia	16 (0.3)	4.50 (2.75-7.35)	1.86 (1.14-3.06)
OSA	88 (1.6)	21.14 (17.11-26.13)	6.70 (5.38-8.35)
Sepsis			
No insomnia or OSA	13 456 (0.3)	1 [Reference]	1 [Reference]
Insomnia	70 (1.5)	4.70 (3.71-5.94)	2.66 (2.10-3.37)
OSA	75 (1.3)	4.37 (3.48-5.48)	2.65 (2.11-3.34)
Shock			
No insomnia or OSA	3264 (0.1)	1 [Reference]	1 [Reference]
Insomnia	20 (0.4)	5.58 (3.60-8.67)	3.61 (2.31-5.63)
OSA	19 (0.3)	4.60 (2.93-7.22)	3.36 (2.13-5.31)
Air or thrombotic embolism			
No insomnia or OSA	1969 (0.1)	1 [Reference]	1 [Reference]
Insomnia	16 (0.3)	7.41 (4.53-12.12)	3.72 (2.36-6.13)
OSA	20 (0.4)	8.03 (5.17-12.47)	3.25 (2.07-5.08)
Hysterectomy			
No insomnia or OSA	3368 (0.1)	1 [Reference]	1 [Reference]
Insomnia	15 (0.3)	4.06 (2.45-6.75)	3.02 (1.81-5.04)
OSA	17 (0.3)	3.99 (2.48-6.43)	2.46 (1.52-3.98)
Ventilation			
No insomnia or OSA	1403 (0.0)	1 [Reference]	1 [Reference]
Insomnia	23 (0.5)	14.92 (9.88-22.53)	6.29 (4.11-9.63)
OSA	95 (1.7)	52.72 (42.83-64.89)	25.29 (20.11-31.80)

Abbreviations: ARR, adjusted relative risk; NA, not applicable; OSA, obstructive sleep apnea; RR, relative risk.

^a^ Severe morbidity was defined by the Centers for Disease Control and Prevention based on *International Classification of Diseases, Ninth Revision (ICD-9)* and *International Statistical Classification of Diseases and Related Health Problems, 10th Revision (ICD-10)* diagnostic and procedure codes. Data were withheld for the following morbidities due to small numbers: acute myocardial infarction, aneurysm, amniotic fluid embolism, cardiac arrest and/or ventricular fibrillation, conversion of cardiac rhythm, heart failure or arrest during surgery or procedure, severe anesthesia complications, sickle cell disease with crisis, and temporary tracheostomy.

^b^ Adjusted for maternal age, payer, educational level, prepregnancy body mass index, parity, country of birth, diabetes, chronic hypertension, gestational hypertension, perinatal depression, and tobacco smoking during pregnancy.

The results of the analysis of risk of IPD and severe morbidity among patients with insomnia compared with those with OSA (rather than with those without a disorder) are described in eTables 2 and 3 in Supplement 1. Among people with either sleep disorder, those with insomnia had reduced risk of any preeclampsia (ARR, 0.75; 95% CI, 0.66-0.84), including preeclampsia with severe features (ARR, 0.76; 95% CI, 0.66-0.88) and without severe features (ARR, 0.70; 95% CI, 0.57-0.87), compared with those with OSA. The risk was not numerically different for any IPD, placental abruption, SGA birth, or preterm birth.

## Discussion

### Principal Findings

In this population-based study of singleton live births in California, we evaluated the risk of IPD and SM in pregnant patients with insomnia or OSA compared with that in those without insomnia or OSA and across diagnostic groupings. We found that insomnia, like OSA, was associated with an increased risk of IPD, SM, and preterm birth. Notably, the risk for birth of an SGA neonate, 1 of the 3 components of IPD, was greater for patients with insomnia than it was for those with OSA compared with those with neither sleep disorder. While our results indicated that OSA was associated with greater overall risk of SM than was insomnia, a higher risk for certain morbidities in patients with insomnia, including disseminated intravascular coagulation, stroke, sepsis, embolism, and hysterectomy, was observed.

### Results in Context

The association between OSA and adverse pregnancy outcomes has been the focus of much research in sleep disorders in pregnancy, with less attention paid to the risks associated with insomnia.^4,27,28^ While our findings of associations between insomnia and the syndrome of IPD are novel, other studies have suggested that insomnia is associated with increased risk for preterm birth^7^ and neonatal morbidity.^29^ In an observational study of nearly 3 million pregnant people in California from 2007 to 2012, insomnia was associated with overall increased risk of preterm birth compared with no sleep disorder diagnoses (14.1% vs 10.9%; RR, 1.3; 95% CI, 1.0-1.7).^7^ These data align with our findings, although we found that the risk of preterm birth was higher in people with insomnia than in those with OSA. We also found that the risk of preterm birth at less than 28 weeks’ and at 28 to less than 32 weeks’ gestation was higher in patients with insomnia than in patients with no sleep disorders.

A prior meta-analysis found that sleep disorders, including insomnia, were associated with risk of birth of neonates large for gestational age, which is in contrast to our findings.^30^ That metanalysis, which included 20 studies with 58 123 250 pregnant people, evaluated sleep disturbances, including poor sleep quality, extreme sleep duration, insomnia symptoms, restless leg syndrome, subjective sleep-disordered breathing, and diagnosed OSA. The authors found that the composite exposure was associated with increased odds for birth of a neonate large for gestational age (odds ratio, 1.40; 95% CI, 1.11-1.77). These conflicting findings are likely explained by the heterogeneity of included studies. We were not able to evaluate the association between comorbid insomnia and OSA in this study’s dataset given the sample size, underscoring the need for further study in large, well-characterized datasets.

We also found that insomnia was associated with increased risk for SM, a finding that aligns with results of a cross-sectional study of 47 million delivery hospitalizations that used the 2006 to 2017 National Inpatient Sample.^15^ The authors of that study found that the prevalence of non–blood transfusion SM was 3.6 times higher for patients with insomnia compared with people without insomnia. In our study, the risk of non–transfusion SM was 2.74 times higher in patients with vs without insomnia, but the prevalence of this complication was 4.8% among those with insomnia in our study compared with 2.4% in the other study.

### Clinical Implications

The rates of insomnia and OSA in the cohort in the present study were lower than those reported in other populations. Overall, OSA is present in 20% of all pregnancies, but the rates vary by trimester, with increasing prevalence rates throughout pregnancy.^27^ A large meta-analysis of over 15 000 patients reported that the prevalence of insomnia during pregnancy was 38%, with rates increasing with gestational age.^20^ The likely explanation for this discrepancy is that many pregnant people with sleep disorders of pregnancy may not report their symptoms to their obstetric clinicians (who also may not be inquiring about them). As these sleep disorders are associated with risk of hypertensive disorders of pregnancy, gestational diabetes, and cesarean delivery, our data underscore the importance of identifying these sleep disorders during pregnancy.

There are various proposed mechanisms for the development of adverse pregnancy outcomes in patients with sleep disorders. One proposed mechanism is that sleep disorders activate the sympathetic nervous system due to circadian disruptions, resulting in poor placental perfusion.^30^ Other studies suggest that sleep disturbances are due to an impaired hypothalamic-pituitary-adrenal axis, which increases inflammation and oxidative stress.^29,31^ Given the known associations between poor placental perfusion and IPD, these are plausible mechanisms that may underlie the association between poor sleep and placentally mediated complications.^11,12^ The results of this study suggest overlapping but unique risk profiles associated with OSA and insomnia. While these findings potentially shed some light on the mechanisms that underlie the association between insomnia and adverse pregnancy outcomes, further study to characterize the mechanisms that drive obstetric risk associated with insomnia are needed.

In our study, insomnia and OSA were associated with distinct demographic profiles. Pregnant people with insomnia were more likely than those with OSA to be underweight and had higher rates of depression and tobacco smoking during pregnancy, associations that previous studies have also identified.^20,31^ The associations between insomnia and birth of an SGA neonate and some other placentally mediated complications suggest that additional fetal monitoring should be considered in pregnancies complicated by insomnia. In addition to routine care, it may be prudent to perform a third-trimester fetal growth assessment as a strategy to identify growth restriction.

While interventions to treat OSA, such as continuous positive airway pressure, are widely used in pregnancy, insomnia treatment may be uderused.^32^ The first-line treatment for insomnia is cognitive behavioral therapy for insomnia (CBT-I), but sleep hygiene, avoidance of stimulants such as caffeine, and pharmacologic therapies are also available.^33^ The use of CBT-I may be effective based on the results of recent clinical trials in pregnant populations. A recent randomized clinical trial found that patients receiving CBT experienced higher rates of remission of insomnia compared with a control group.^34^ Pharmacologic agents, such as antidepressants, melatonin, trazodone, zolpidem, and over-the-counter medications, such as diphenhydramine, may also be considered and are thought to be safe in pregnancy.^35^

Data on the long-term effects of insomnia on both birthing people and their infants is lacking. The association of fetal IPD exposure with long-term behavioral outcomes has been evaluated,^11^ but further studies are needed to evaluate whether treatment of insomnia can mitigate these risks. SM in pregnancy affects long-term health and burdens the health care system. More work is needed to investigate the mechanism by which insomnia is associated with increased risk of certain subtypes of SM.

### Strengths and Limitations

There are several strengths of this study. We used a large population-based sample, which increases the study’s generalizability. We included data about OSA to facilitate comparisons about the magnitude of associated risks. Additionally, we included a reference group of pregnant people who did not have insomnia or OSA for analyses, whereas some insomnia studies have included people without insomnia as the reference group. Although not the study’s primary goal, we also compared the risk of IPD and SM between people with insomnia and people with OSA. As such, our analyses are robust and allow comparisons of the magnitude of risks associated with insomnia vs OSA.

This study also has limitations, some of which come from a reliance on *ICD-9* and *ICD-10* codes. To our knowledge, the sensitivity and specificity for the presence or absence of maternal insomnia and OSA have not been reported. In this retrospective analysis, we could not assess the temporal nature or severity of the sleep disorder diagnoses in pregnancy. For example, we were unable to assess whether the risk of insomnia increased as pregnancy progressed or whether there may be differences in magnitude of risk between mild and severe forms of insomnia. Recent studies have shown temporal trends of sleep disorders in pregnancy, with more severe symptoms early in pregnancy leading to worse outcomes for the birthing parent and neonate.^21,36^ We did not have information on treatment of sleep disorders in the study population, and we could not evaluate whether treatment impacted risk of adverse outcomes, as this was an observational study. It is possible that treatment of insomnia and OSA may decrease the risk of adverse pregnancy outcomes, and adherence to and duration of treatment may impact these risks. The prevalence of insomnia and OSA has increased in modern cohorts; this is likely to due to increased ascertainment and awareness among clinicians. The contribution of treatment and temporal trends was a potential source of bias in this study. We also had insufficient numbers to evaluate the risks associated with comorbid insomnia and sleep apnea in pregnancy, an important area of emerging research. In addition, we limited our analysis to live births. We acknowledge that sleep disorders may be associated with adverse fetal outcomes, such as stillbirth or miscarriage, and future studies using more comprehensive datasets including early pregnancy losses and fetal deaths are warranted to evaluate these important associations.

## Conclusions

In this cross-sectional study of singleton live births in California, we found that insomnia and OSA were associated with increased risk for IPD and SM. Comparisons with individuals without sleep disorders indicated that insomnia vs OSA was associated with higher risk of certain morbidities and complications, including placental abruption, SGA birth, and preterm birth at less than 32 weeks’ gestation, as well as several severe complications, including disseminated intravascular coagulation, stroke, and hysterectomy. Our findings underscore the need for further studies to identify best practices around screening and treatment for insomnia and randomized studies to evaluate the impact of insomnia treatment in prevention of adverse outcomes. Increased recognition of insomnia as a potentially modifiable risk factor for obstetric complications is needed.

## Notes

Supplement 1. **eTable 1.** Source of Diagnoses in Birth Certificate and HCAI Records **eTable 2.** Risk of Ischemic Placental Disease and Preterm Birth Among People With Insomnia Compared With People With Obstructive Sleep Apnea **eTable 3.** Risk of Severe Morbidity Among People With Insomnia Compared With People With Obstructive Sleep Apnea

Supplement 2.
Data Sharing Statement
