# Supplementary material for: Ischemic Placental Disease and Severe Morbidity in Pregnant Patients With Sleep Disorders
Source: JAMA Netw Open. 2025 Sep 16;8(9):e2532189. doi: 10.1001/jamanetworkopen.2025.32189 (PMC12441874; doi:10.1001/jamanetworkopen.2025.32189)
Supplement: Supplement 1. — eTable 1. Source of Diagnoses in Birth Certificate and HCAI Records eTable 2. Risk of Ischemic Placental Disease and Preterm Birth Among People With Insomnia Compared With People With Obstructive Sleep Apnea eTable 3. Risk of Severe Morbidity Among People With Insomnia Compared With People With Obstructive Sleep Apnea [file jamanetwopen-e2532189-s001.pdf]

Supplementary Online Content

Ross N, Baer RJ, Oltman SP, et al. Ischemic placental disease and severe morbidity in pregnant patients with sleep disorders. *JAMA Netw Open*. 2025;8(9):e2532189. doi:10.1001/jamanetworkopen.2025.32189

eTable 1. Source of Diagnoses in Birth Certificate and HCAI Records
eTable 2. Risk of Ischemic Placental Disease and Preterm Birth Among People With Insomnia Compared With People With Obstructive Sleep Apnea
eTable 3. Risk of Severe Morbidity Among People With Insomnia Compared With People With Obstructive Sleep Apnea

This supplementary material has been provided by the authors to give readers additional information about their work.

© 2025 Ross N et al. *JAMA Network Open*.

eTable 1
Source of diagnoses in birth certificate and HCAI records

	ICD-9	ICD-10	Birth certificate indication
Insomnia	780.52, 307.41, 307.42, 327.00, 292.85, V69.4	F51.01, F51.02, F51.03, F51.04, F51.05, F51.09, G47.01, G47.02, G47.00, Z72.821,	
Obstructive sleep apnea	780.57, 326.23	G47.30, G47.33	
Preeclampsia	642.4, 642.5, 642.6, 642.7	O11, O14.0, O14.1, O14.2, O14.9, O15	X
Preeclampsia with severe features	642.5, 642.6, 642.7	O11, O14.1, O14.2, O15	X
Preeclampsia without severe features	642.4	O14.0, O14.9	
Placental abruption	641.2, Infant 762.1	O45, Infant P02.1	
Cesarean delivery	669.7, Procedure code: 74.0, 74.1, 74.2, 74.99, Infant 763.4	O82, Procedure code: 10D00Z0, 10D00Z1, 10D00Z2, Infant P03.4	X
Vaginal delivery			X
Smoked	649.0, 305.1	Z72.0, F17.2, Infant P04.2	X
Chronic hypertension without preeclampsia	642.0, 642.1, 642.2	O10, O16, Infant P00.0	X
Gestational hypertension without preeclampsia	642.3	O13	
Preexisting diabetes	249, 250, 648.0	E10, E11, E12, E13, E14, O24.0, O24.1, O24.2, O24.3, Infant P70.1	X
Gestational diabetes	249, 250, 648.0, 648.8	E10, E11, E12, E13, E14, O24.0, O24.1, O24.2, O24.3, O24.4, O24.9 Infant P70.0, P70.1	X
Depression	296.2, 296.3, 311	F32.0, F32.1, F32.2, F32.3, F32.8, F32.9, F33.0, F33.1, F33.2, F33.3, F33.4, F33.8, F33.9	

© 2025 Ross N et al. *JAMA Network Open*.

eTable 2
Risk of ischemic placental disease and preterm birth among people with insomnia compared to people with obstructive sleep apnea

	Insomnia<br>n (%)<br>RR (95% CI)<br>aRR (95% CI)	OSA<br>n (%)<br>RR (95% CI)<br>aRR (95% CI)
**Sample**	4,783	5,642
**No IPD/PTB**	3,377 (70.6)	3,794 (67.3)<br>*Reference*
**Any IPD**[a]	1,406 (29.4)<br>0.89 (0.84, 0.96)<br>0.97 (0.89, 1.05)	1,848 (32.8)
Any PEC[a]	492 (10.3)<br>0.56 (0.50, 0.62)<br>0.75 (0.66, 0.84)	1,126 (20.0)
PEC with severe features[a]	357 (7.5)<br>0.51 (0.46, 0.59)<br>0.76 (0.66, 0.88)	857 (15.2)
PEC without severe features[a]	168 (3.5)<br>0.59 (0.49, 0.72)<br>0.70 (0.57, 0.87)	329 (5.8)
Placental abruption	97 (2.0)<br>1.07 (0.81, 1.41)<br>0.95 (0.68, 1.31)	102 (1.8)
SGA birth[b]	497 (10.4)<br>1.35 (1.19, 1.55)<br>1.12 (0.96, 1.31)	397 (7.0)
Preterm birth (<37 weeks)	711 (14.9)<br>0.93 (0.84, 1.03)<br>1.04 (0.93, 1.17)	870 (15.4)
<28 weeks	47 (1.0)<br>0.96 (0.65, 1.42)<br>1.12 (0.72, 1.76)	55 (1.0)
28 0/7–31 6/7 weeks	93 (1.9)<br>1.08 (0.81, 1.43)<br>1.16 (0.83, 1.62)	97 (1.7)
32 0/7–33 6/7 weeks	96 (2.0)<br>0.82 (0.63, 1.06)<br>0.91 (0.67, 1.24)	133 (2.4)
34 0/7–<37 weeks	475 (9.9)<br>0.92 (0.82, 1.04)<br>1.04 (0.90, 1.20)	585 (10.4)

[a]Not adjusted for HTN disorders
[b]Birth weight <10th percentile per Talge 2014.
HDP: hypertensive disorders of pregnancy; IPD: ischemic placental disease; OSA: obstructive

© 2025 Ross N et al. *JAMA Network Open*.

eTable 3
Risk of severe morbidity among people with insomnia compared to people with obstructive sleep apnea

	Insomnia<br>n (%)<br>RR (95% CI)<br>aRR (95% CI)	OSA<br>n (%)<br>RR (95% CI)<br>aRR (95% CI)
**Sample**	4,783	5,642
**No SM[a]**	4,417 (92.4)	5,097 (90.3)<br>*Reference*
**Any SM**	366 (7.7)<br>0.79 (0.69, 0.90)<br>0.80 (0.68, 0.93)	545 (9.7)
SM without blood transfusions	231 (4.8)<br>0.69 (0.59, 0.81)<br>0.71 (0.58, 0.85)	394 (7.0)
**Acute renal failure**	28 (0.6)<br>0.61 (0.39, 0.97)<br>0.56 (0.32, 0.96)	53 (0.9)
**Acute respiratory distress syndrome**	50 (1.1)<br>0.68 (0.48, 0.97)<br>0.68 (0.45, 1.03)	85 (1.5)
**Disseminated intravascular coagulation**	32 (0.7)<br>1.09 (0.67, 1.76)<br>1.02 (0.58, 1.79)	34 (0.6)
**Blood transfusion**	142 (3.0)<br>0.93 (0.75, 1.17)<br>0.93 (0.71, 1.21)	175 (3.1)
**Eclampsia**	26 (0.5)<br>0.86 (0.52, 1.43)<br>0.67 (0.37, 1.21)	35 (0.6)
**Puerperal cerebrovascular disorders**	19 (0.4)<br>1.56 (0.78, 3.12)<br>1.51 (0.67, 3.36)	14 (0.3)
**Pulmonary edema/ acute heart failure**	16 (0.3)<br>0.21 (0.12, 0.36)<br>0.25 (0.14, 0.45)	88 (1.6)
**Sepsis**	70 (1.5)<br>1.07 (0.78, 1.49)<br>0.82 (0.56, 1.22)	75 (1.3)
**Shock**	20 (0.4)<br>1.21 (0.65, 2.27)<br>1.08 (0.51, 2.23)	19 (0.3)
**Air and thrombotic Embolism**	16 (0.3)<br>0.92 (0.48, 1.78)<br>1.91 (0.91, 4.02)	20 (0.4)
**Hysterectomy**	15 (0.3)<br>1.02 (0.51, 2.04)<br>1.05 (0.46, 2.38)	17 (0.3)
**Ventilation**	23 (0.5)<br>0.28 (0.18, 0.45)<br>0.29 (0.17, 0.49)	95 (1.7)

© 2025 Ross N et al. *JAMA Network Open*.

[a]Severe morbidity defined to by US Centers for Disease Control and Prevention based on ICD diagnostic and procedure codes.
Data withheld for the following morbidities due to small numbers**:** acute myocardial infarction, aneurysm, amniotic fluid embolism, cardiac arrest / ventricular fibrillation, conversion of cardiac rhythm, heart failure/arrest during surgery or procedure, severe anesthesia complications, sickle cell disease with crisis, and temporary tracheostomy
CI: confidence interval; OSA: obstructive sleep apnea: RR: relative risk; SM: severe morbidity as defined by the CDC.
