# Supplementary material for: Ischemic Placental Disease and Severe Morbidity in Pregnant Patients With Sleep Disorders
Source: JAMA Netw Open. 2025 Sep 16;8(9):e2532189. doi: 10.1001/jamanetworkopen.2025.32189 (PMC12441874; doi:10.1001/jamanetworkopen.2025.32189)
Supplement: Supplement 2. — Data Sharing Statement [file jamanetwopen-e2532189-s002.pdf]

Data Sharing Statement

Ross. Ischemic Placental Disease and Severe Morbidity in Pregnant Patients With Sleep Disorders. *JAMA Netw Open*. Published September 16, 2025.
doi:10.1001/jamanetworkopen.2025.32189

Data
Data available: No
